# Lateral alveolar ridge augmentation procedure using subperiosteal tunneling technique: a pilot study

**DOI:** 10.1186/s40902-018-0142-8

**Published:** 2018-02-25

**Authors:** Ashish Kakar, Kanupriya Kakar, Bappanadu H. Sripathi Rao, Annette Lindner, Heiner Nagursky, Gaurav Jain, Aditya Patney

**Affiliations:** 1 Yenepoya University Dental College, University Road, Mangalore, 575018 India; 2 Private Practice, New Delhi, India; 3 Institute for Clinical Chemistry and Laboratory Medicine, Medical Center, University of Freiburg, HugstetterStrasse 55, 79106 Freiburg, Germany; 4 Dental Surgery, Indraprastha Apollo Hospitals, New Delhi, India; 5 Mahajan Imaging Center, New Delhi, India

**Keywords:** Alveolar, Ridge, Augmentation, Subperiosteal, Tunneling, Alloplastic

## Abstract

**Background:**

In this research article, we evaluate the use of sub-periosteal tunneling (tunnel technique) combined with alloplastic in situ hardening biphasic calcium phosphate (BCP, a compound of β-tricalcium phosphate and hydroxyapatite) bone graft for lateral augmentation of a deficient alveolar ridge.

**Methods:**

A total of 9 patients with deficient mandibular alveolar ridges were included in the present pilot study. Ten lateral ridge augmentation were carried out using the sub-periosteal tunneling technique, including a bilateral procedure in one patient. The increase in ridge width was assessed using CBCT evaluation of the ridge preoperatively and at 4 months postoperatively. Histological assessment of the quality of bone formation was also carried out with bone cores obtained at the implant placement re-entry in one patient.

**Results:**

The mean bucco-lingual ridge width increased in average from 4.17 ± 0.99 mm to 8.56 ± 1.93 mm after lateral bone augmentation with easy-graft CRYSTAL using the tunneling technique. The gain in ridge width was statistically highly significant (*p* = 0.0019). Histomorphometric assessment of two bone cores obtained at the time of implant placement from one patient revealed 27.6% new bone and an overall mineralized fraction of 72.3% in the grafted area 4 months after the bone grafting was carried out.

**Conclusions:**

Within the limits of this pilot study, it can be concluded that sub-periosteal tunneling technique using in situ hardening biphasic calcium phosphate is a valuable option for lateral ridge augmentation to allow implant placement in deficient alveolar ridges. Further prospective randomized clinical trials will be necessary to assess its performance in comparison to conventional ridge augmentation procedures.

## Background

Atrophic alveolar ridges may impede the proper placement of dental implants in their appropriate positioning necessary to guarantee their long-term function and an acceptable esthetic profile of the final prosthesis [1, 2]. The use of autogenous bone for the reconstruction and augmentation of such ridges to facilitate implant rehabilitation has traditionally been the gold standard in this area [3]. However, use of autogenous bone carries the obvious drawbacks of additional donor site surgery and its attendant sequelae and the resultant reduced patient acceptance. The last few decades have seen a proliferation of research into bone graft substitutes to autogenous bone. Several materials have been described and promoted as alternatives to the autogenous bone harvest [4].

Calcium phosphates have been amongst the earliest of the potential bone graft substitute materials to be identified, based upon their similarity to the chemical structure of the inorganic components of native bone [5] which is largely composed of crystalline hydroxyapatite form of calcium phosphate. Currently, the most widely used forms of synthetic calcium phosphate are the resorbable β-tricalcium phosphates (β-TCP), the virtually non-resorbing crystalline hydroxyapatite (HA), and a combination of the two phases called biphasic calcium phosphates (BCP, a compound of β-tricalcium phosphate and hydroxyapatite).

Calcium phosphate materials are osteoconductive in nature and act to guide bone formation towards the center of the graft. While the β-tricalcium phosphate resorbs completely and is replaced with new bone, hydroxyapatite remains unresorbed but integrates into newly forming host bone [4, 6–8].

GUIDOR *easy-graft* CRYSTAL (Sunstar Suisse SA, Etoy, Switzerland) is a biphasic synthetic, in situ hardening bone graft substitute composed of 60% HA and 40% β-TCP [9]. The presence of HA causes the material to persist after new bone formation, integrating into the newly formed bone, while facilitating volume maintenance during initial healing. The material is directly applied from an applicator syringe, it is moldable, and, upon contact with blood, i.e. after application into the bone defect, hardens into a porous, stable scaffold. This in situ hardening property is particularly well suited to subperiosteal augmentation techniques. It allows this material to be readily inserted into the defect through the syringe and further molded and modeled into the desired shape through the gingiva during a short period after application before harden. The hardened scaffold remains stable during the early phases of healing [10].

Restoration of bone volume in patients with atrophic ridges is a prerequisite to esthetic and functional implant-supported prosthetic rehabilitation [11]. Several techniques to achieve enhanced bone volume have been described, including ridge splitting, alveolar distraction osteogenesis, guided bone regeneration (GBR) techniques, cortico-cancellous block onlay, and interpositional grafts, etc. [12–16]. Onlay grafting seems to be the most predictable of these techniques. However, in addition to the invasiveness of some of these techniques, there are also the complications of partial or complete graft loss on account of postoperative wound dehiscence and graft exposure, especially with onlay graft techniques and later graft resorption during the consolidation phase of the cortico-cancellous block grafts [17].

The risk of bone exposure and loss is increased with the use of crestal full thickness incisions for surgical access; implants which were placed without flap elevation showed significantly greater amount of osseointegration and the bone height around the implants as compared to the implants placed with flap elevation [18]. Flap management for block grafting necessitates surgical wound closure directly over the bone graft. Hence, any wound or suture dehiscence at the wound margins of the crestal incision immediately results in exposure of the graft, increasing the possibility of contamination and failure. Recent reports have suggested a modification of the surgical approach to minimize the risk of graft exposure as a complication of onlay bone grafting [18]. The subperiosteal augmentation techniques involve placement of vertical incisions some distance away from the actual site of bone graft placement. The augmentation site is approached through the creation of a subperiosteal tunnel. This allows the graft material to be placed under intact mucoperiosteal tissue, thereby minimizing the risk of exposure of the graft on account of wound dehiscences.

In the present report, we present a series of 9 patients with a total of 10 lateral augmentation of deficient alveolar ridges using graft material applied through the subperiosteal tunneling approach. To our knowledge, this is the first systematic report investigating the effectiveness of this graft to augment atrophic alveolar ridges in combination with the subperiosteal tunneling technique.

## Methods

### Study design

Nine consecutive patients between 18 and 78 years of age, with resorbed mandibular posterior ridges subsequent to tooth extraction who met the study inclusion criteria, were included. In total, 10 lateral ridge augmentation were performed using in situ hardening biphasic calcium phosphate graft material using the subperiosteal tunneling approach.

#### Inclusion criteria

Patients who presented with edentulous lateral mandible with at least the second premolar, and the first and second molars missing and horizontally atrophied alveolar crest that impairs prosthodontic rehabilitation were included in the study.

#### Exclusion criteria

Patients with severe alveolar ridge deficiencies (> 4 mm) as compared to the dentate segments, recent extractions (within 6 months), and patients with residual root stumps in the site, or periapical lesions, and infections or abscesses adjacent to prospective surgical sites were excluded from the study. Similarly, pregnant or lactating women, patients with known systemic diseases or metabolic disorders (e.g., HIV and diabetes) or those on medications known to be detrimental to bone healing (e.g., treatment with bisphosphonates and steroids), smokers or smokeless tobacco users, alcohol and psychotropic drug abusers, and patients with a history of malignant or other diseases treated with radiotherapy, or with chemotherapy within the last 5 years, were also excluded.

A signed, written informed consent was sought from each participant only after a full explanation had been given, a patient information leaflet offered, and time allowed for consideration. The right of the patient to refuse to participate in the clinical study without giving reasons was respected.

### Surgical procedure

**Fig. 1:**
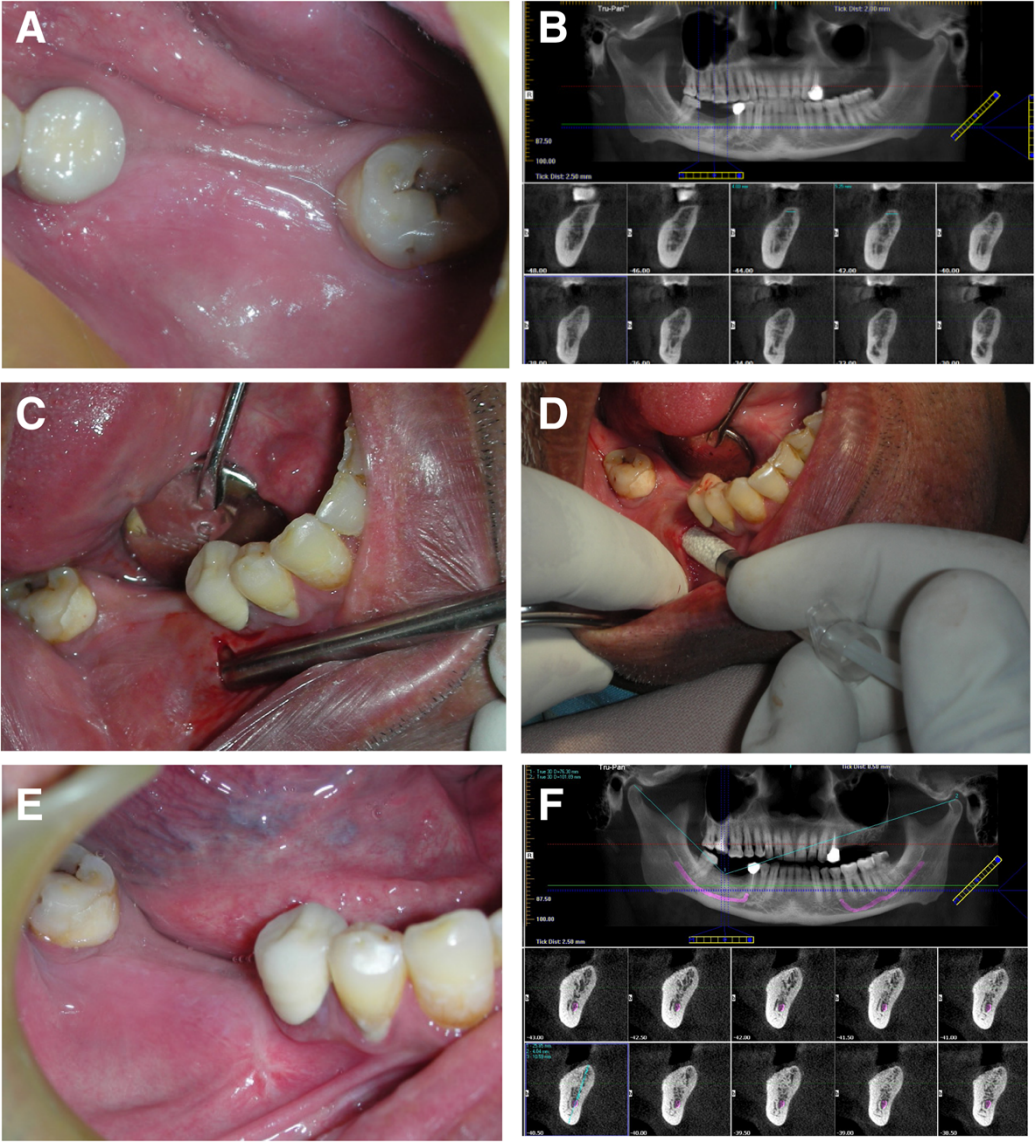
**a** Clinical situation with missing mandibular molars. **b** Preoperative CBCT showing deficient alveolar ridge width. **c** 5-mm vertical incision made for tunnel preparation and periosteal dissection for the tunnel. **d** Easy-graft CRYSTAL being injected into the prepared tunnel. **e** Postoperative healing. Notice the minimal area of scarring at the vertical incision site. **f** Postoperative CBCT showing increased ridge width and graft consolidation

Before scheduling the surgery, a preoperative CBCT scan was performed to assess the ridge atrophy at the edentulous site and to decide if the patient fulfills the inclusion criteria. The surgical procedure was performed under local anesthesia with Lidocaine HCl 2% (Lignospan Special, Septodont, France) with adrenaline 1: 80,000. Figure 1a, b represents the initial CBCT and clinical presentation of one case.

A 5-mm vertical incision was made 8–10 mm away from the site to be augmented (Fig. 1c). The augmentation was performed with Tunnel Control instrument set (Hager & Meisinger, Neuss, Germany). A primary tunnel (pouch) was formed by detaching the periosteum from the underlying bone with the raspatorium supplied with the instrument set. Next, the cortical bone was partially removed in the tunnel, forming a furrow in the bone surface, exposing the underlying cancellous bone structures in the tunnel. This was achieved with a water-cooled round bur protected towards the soft-tissue component of the tunnel (supplied in the Tunnel Control instrument set). The bone graft substitute was prepared in the syringe according to the manufacturer’s instructions for use. The material was inserted into the tunnel (Fig. 1d), formed into the desired shape in situ, and pressed against the bone for 1–2 min by the surgeon. In contact with blood, the material hardened within this time span into a solid, porous body. Approximately, two to three applications of graft material 0.4 ml were used (1.2 ml per augmented site), as needed to restore the shape of the alveolar ridge. The wound was closed using an interrupted 4-0 vicryl suture. Figure 3a shows postoperative healing after 3 weeks of the grafting procedure.

### CBCT scanning

**Fig. 2:**
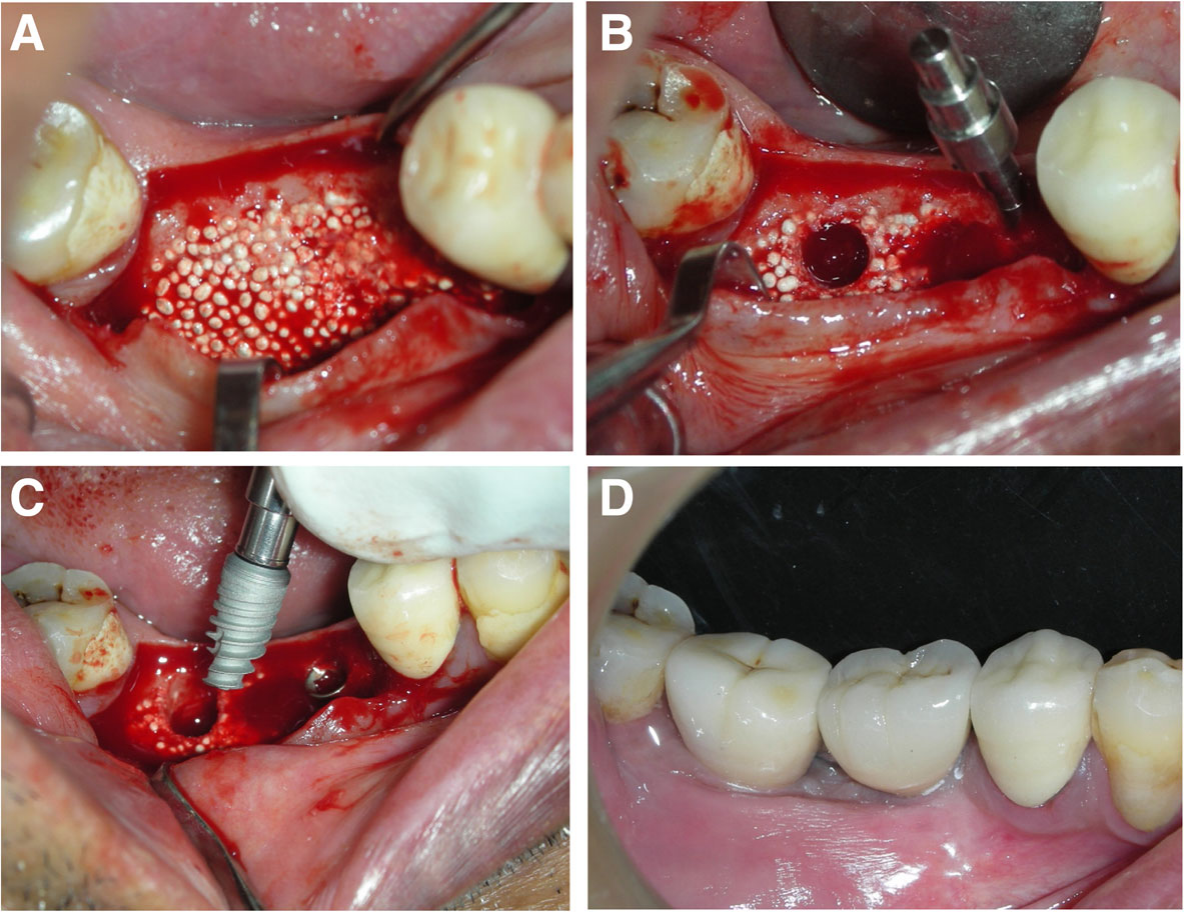
**a** Mucoperiosteal flap reflection post healing and graft consolidation showing the graft well integrated into the native bone and an increased width of the alveolar ridge. **b** Two core biopsy samples harvested from the augmented area. Anterior implant preparation was done. **c** Implant insertion into the site. Note that a wide-diameter implant was placed into the second molar area. **d** Two individual implant crowns were placed as the final prosthesis

Cone beam computer tomography (CBCT) scans were performed preoperatively and at 4 months post operatively to assess the gain in horizontal dimensions of the ridge. CBCT scanning was performed using a K9500 CBCT scanner (Kodak Dental Systems, Carestream Health Inc. Rochester, NY). For the present study, the medium mode with 15 × 9 cm field of view was used and the spatial resolution of each voxel was 0.2 mm^3^. Dental image processing was performed with interactive CT/CBCT image processing software (3D™ Cyber-Med Inc., Seoul, South Korea). The augmented volume was estimated by CBCT using the image processing software, graft outlines were marked manually on the scans. All augmented sites were treated for Dental implant placement. At the healing phase, the area was re-entered after reflection of a full thickness muco-periosteal flap (Fig. 2a). Dental implants were placed in the grafted area (Fig. 2b, c). In one patient, a core biopsy was obtained using a trephine drill (Komet Dental, Germany) in a bucco-lingual direction to harvest the tissue sample for histo-morphometric analysis. Figure 2d shows the final prosthetic rehabilitation done with individual crowns in the augmented sites.

### Histological preparation and histomorphometric analysis

**Fig. 3:**
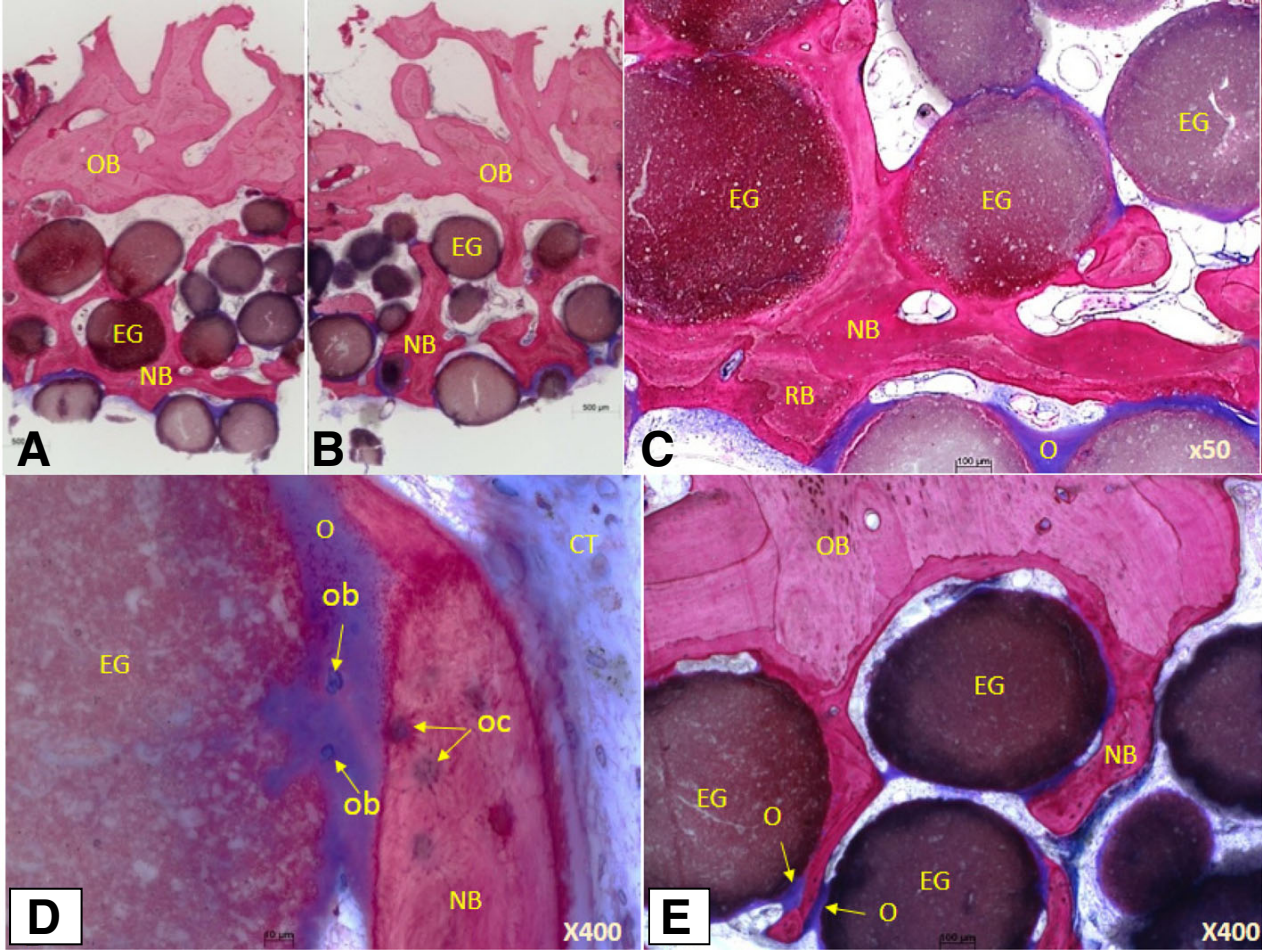
**a**, **b** Saw cuts of the core biopsy are shown in the upper parts of the original bone; the lower parts represent the augmented area. Here, some easy-graft particles are partially or completely embedded in osteoid or newly formed bone. New bone with integrated biomaterial forms dense trabeculae and exhibits a mature, lamellar structure. Interosseous and intergranular connective tissue is well perfused and free of inflammation (see also histomorphometric data and details). Newly formed bone (NB) is stained (dark magenta), original older bone (OB; light magenta), easy-graft (EG; dark brown), and soft tissue (light blue) (undecalcified ground sections stained with azure II and pararosaniline; overviews are compilations of several single photos, original magnification ×  50). **b** For histomorphometric purpose were highlighted: easy-graft granules (light blue), osteoid (dark blue), and newly formed bone (red). The yellow line limits the region of interest. Tissue area under this line is set equal to hundred percent. **c** Graft particles (EG) tightly integrated in newly formed mature bone (NB) or blue-stained osteoid (O); different staining characteristics of newly formed bone and recently formed bone (RB). **d**, **e** Graft particles (EG) completely integrated in newly formed mature bone (NB) or broad seams of osteoid (O) including osteoblasts (ob); new bone (NB) is added to original bone (OB) displaying entrapped osteocytes (oc); densely vascularized connective tissue (CT); no signs of inflammation osteoid setting. *Easy-graft* particle (EG), newly formed bone (NB), recently formed bone (RB), osteoid (O), original bone (OB), osteoblast (ob), osteocyte (oc), connective tissue (CT)

Histological sections were prepared from a bone core biopsy obtained in an oblique direction from the buccal cortex in one patient at the site of subsequent implant placement. Sample fixation for tissue samples were done in 4% buffered formaldehyde for at least 1 week at 4 °C before shipping to the histology lab. Qualitative, histomorphometric evaluation were performed of histological sections of two specimens from the same patient. Qualitative evaluation assessed bone formation (woven and lamellar bone), implant resorption, and presence of fibrous tissue and assessed cellular infiltration and type of cells, and finally, quantitative evaluation included histomorphometric measurements of percentage new bone, fibrous tissue, and remaining bone graft substitute using a special software program (Qwin, Quips, Leica, Glattbrugg, Switzerland). Figure 3a–e shows the results of the core biopsy. The graft material particles were tightly integrated into the newly formed bone. The area was also rich in osteoid tissue with osteoblasts and osteocytes. There was no inflammatory tissue present in the analyzed tissue, and the matrix was densely vascularized.

### Statistics

Data were expressed as means ± standard deviations (SD). A non-parametric Wilcoxon rank-sum test was used to assess significance of intra-group longitudinal changes for ridge width changes as measured by CBCT analysis.

This study had been approved by IRB (Approval Number SHRAB/DS/2012).

## Results

A total of 9 patients who fulfilled the inclusion criteria were enrolled in the study, and a total 10 ridge augmentations were performed using the subperiosteal tunneling technique with an alloplastic in situ hardening bone graft substitute. One patient underwent a bilateral procedure. There were 5 males and 4 females, in an age range between 49 and 78 years (median age 55 years). Wound healing was assessed qualitatively in terms of postoperative pain, swelling, hematoma formation, incidence of wound dehiscence, and graft loss. The surgical sites healed uneventfully, without any intra-operative or postoperative complications in any of the patients. All patients who received bone augmentation eventually had dental implants placed in the grafted areas.

Ridge width changes 4 mm below the crest measured by CBCT before and 4 months after the augmentation were assessed for all 10 sites (Table 1). The preoperative mean bucco-lingual ridge width at 4 mm from the crest of the ridge was 4.17 ± 0.99 mm, which increased to 8.56 ± 1.93 mm after lateral bone augmentation with graft material. The average increase of the width ridge was 4.39 ± 1.58 mm and highly significant in the crestal location (*p* < 0.05).

**Table 1:** Ridge width gain at 4 mm from the crest of the ridge

Patient	Gender	Age (years)	Width at 4 mm	
			Pre-op	Post-op
1	M	78	5.01	9.56
2	F	54	3.78	9.25
3	M	49	4.82	7.76
4	F	56	5.70	9.80
5	F	54	4.83	9.45
6	M	77	4.30	11.92
7	M	56	4.28	8.02
8	F	66	3.42	5.50
9	M	50	2.50	5.70
10	M	50	3.02	8.60
Mean		*59 ± 11*	*4.17 ± 0.99*	*8.56 ± 1.93*
*p* value	0.0019			

*Pre-op* preoperative, *Post-op* postoperative

Bone cores were obtained for histomorphometric assessment at the time of dental implant insertion for 1 patient (Fig. 2a–c); the results of which are tabulated in histomorphometric analysis in Table 2. Histomorphometric assessment showed a mean of 27.6% new bone formation at 4 months after the bone grafting, with an overall mineralized fraction being 72.3%. The residual structure comprised of bone forming healthy osteoid tissue and bone marrow as well as some connective tissue. The individual bone graft substitute particles were well integrated into the host bone or osteoid in intimate contact with the particle surface (Fig. 3 a-e). New bone around the graft particles showed a natural ossification sequence with osteoblasts (ob) building up new bone and osteocytes (oc) well embedded in the newly formed bone (Fig. 3c–e).

**Table 2:** Histomorphometric Assessment of Bone Cores from 1 patient

Section	A	B	Mean
Dimension (mm)	4,9 × 3,3	5,0 × 3,4	
Coverage (%)			
New bone	25,6	29,7	27,6
Easy-graft	49,8	39,6	44,7
Mineralized fraction	75,4	69,3	72,3
Osteoid	2,6	3,6	3,1
Amorphous calcified substance	2,4	2,0	2,2
Connective tissue, Bone marrow	19,6	25,1	22,4

## Discussion

The use of bone augmentation prior to dental implant placement should facilitate the formation of good quality bone with minimal loss of bone volume during healing [3]. The ideal technique of bone augmentation should maintain the space during the period of bone ingrowth and implant healing and provide a stable augmentation of the bone. The ideal graft material, moreover, should integrate into native bone, be readily available, and be easy to place with minimal patient morbidity. The usual lateral augmentation techniques with autogenous block grafts require the elevation of extensive, full thickness mucoperiosteal flaps. The risk of wound dehiscence with resultant loss of augmentation volume is a major risk associated with these procedures [10].

The procedure using a vertical incision and creation of a subperiosteal tunnel for access to the deficient bone site, as described here, seems to be well suited to preimplant bone augmentation and has the following advantages:

1. Easy, minimally invasive technique that can be performed under local anesthesia in the dental office
2. Minimal postoperative patient morbidity due to decreased extent of surgical exposure
3. Minimal risk of loss of augmentation volume due to loss of graft arising from local wound dehiscence

The graft material being a biphasic calcium phosphate allows the maintenance of augmentation volume during the consolidation phase but supports the ingrowth of native bone. Also, its moldability and in situ hardening characteristic facilitates its easy placement at the site and provides stability during the critical early healing phase [9]. Control of the surgically expanded soft tissue volume is believed to prevent resorption of graft material over the long term [19, 20].

Block and Degen [17] used the tunneling technique for lateral augmentation in 13 posterior mandibular ridges, using human mineralized particulate bone and followed it up with placement of 35 implants in the grafted sites. They reported an estimated 5 to 8 mm lateral ridge augmentation immediate postoperatively and a subjective maintenance of at least 50% of the augmentation 3 months later. However, no objective, definitive measurements were made in their study. In our investigation for reconstruction of posterior mandibular horizontal defects using a “tunneling technique,” a mean crestal ridge width gain of 4.39 mm was achieved based on radiological CBCT measurements. Hasson [18] reported the use of subperiosteal tunnel technique for augmentation of the maxilla and the mandible. However, no comparative measurements were reported. Kfir et al. [21] used the tunnel technique for guided bone regeneration and reported a vertical gain between 2.4 and 5.1 mm and a ridge width gain of 1.3 to 3.9 mm.

Jeong et al. [16] studied simultaneous flapless implant placement and peri-implant bone grafting through subperiosteal tunneling, an animal study using mongrel dogs, and found significantly better new bone coverage on the exposed threads than the control group, indicating utility of the technique in conjunction with implant placement as well.

In terms of complications, Block and Degen [17] noted small wound dehiscences with graft loss adjacent to the incision in four ridges. This resulted in the ridge being too narrow in the site immediately adjacent to the most anterior tooth, necessitating cantilevered restorations. The same limitation to the procedure was also noted by Hasson [18]. The latter also remarked that it is a partly blind procedure which does not allow visualization of the deficient ridge. Also, the procedure might involve a slightly longer learning curve, with the need for careful and delicate surgical maneuvers and tissue handling.

Hasson [18] emphasized the benefit of decortication prior to graft insertion and the tenting effect produced using collagen membranes. Kfir et al. [21] also used a barrier membrane over the synthetic bone graft substitute and autologous fibrin. In our series of cases, no barrier membranes were used with the synthetic bone graft for achieving clinical success in correction of horizontal bone defects in the atrophic ridge. However, the volume of graft material used was higher than reported by other investigators who used grafts with a barrier membrane [18]. The use of barrier membranes for graft stabilization and space creation for augmentation has been challenged recently by many investigators [22, 23, 24, 25, 26]. Ridge augmentation with xenografts without membranes is also reported to correct both horizontal and vertical defects [23, 24, 27–29]. It is thought that a membrane may inhibit progenitor cell migration and angiogenesis by presenting a physical barrier to chemotaxis [24]. Nevins et al. [24] used three different graft combinations with the minimally invasive tunnel approach. Post healing trephine biopsies from the grafted area were analyzed by micro-CT analysis and with light microscopy. They showed a mean mineralized tissue fraction ranging from 34.6 ± 8.7% to 52.9 ± 12.9%. In our investigation, the histomorphometric analysis showed a mean mineralized fraction of 72.3% (two core biopsy samples from the same subject). New bone formation comprised 27.6% of the grafted area; 44.7% was residual graft material. The total mineralized fraction was 72.3% after 4 months. This is consistent with our earlier results using the same graft material for ridge preservation where 74.34% of mineralization was reported [26]*.* In a recently published study, Lee [30] used a similar subperiosteal tunneling technique for bone augmentation in 60 sites in 21 patients using anorganic bovine bone particles mixed with rhPDGF-BB and no tenting screws, space making devices, or cell-occlusive membranes. The authors also performed a histomorphometric analysis for 1 patient at 6 months after the bone augmentation procedure. The authors reported 50% bone formation, 47% bone marrow or fibrous tissue, 100% vital bone, and no residual bone graft material.

Within the limits of this study, it can be concluded that the subperiosteal tunneling technique in combination with an in situ hardening alloplastic bone graft substitute provides predictable lateral ridge augmentation for implant placement in atrophic ridges while reducing the invasiveness of the procedure.

## Conclusions

The use of bone augmentation prior to dental implant placement should facilitate the formation of good quality bone with minimal loss of bone volume during healing [3]. The ideal technique of bone augmentation should maintain the space during the period of bone ingrowth and implant healing and provide a stable augmentation of the bone. From our present study, the tunneling technique for lateral bone augmentation seems to be a good approach to the procedure. It provides a predictable augmentation through a minimally invasive technique with minimal postoperative complications. Larger, controlled studies maybe designed to further assess the utility of our technique.
